# The Mitochondria-Targeted Antioxidants and Remote Kidney Preconditioning Ameliorate Brain Damage through Kidney-to-Brain Cross-Talk

**DOI:** 10.1371/journal.pone.0051553

**Published:** 2012-12-14

**Authors:** Denis N. Silachev, Nikolay K. Isaev, Irina B. Pevzner, Ljubava D. Zorova, Elena V. Stelmashook, Svetlana V. Novikova, Egor Y. Plotnikov, Vladimir P. Skulachev, Dmitry B. Zorov

**Affiliations:** 1 A.N. Belozersky Institute of Physico-Chemical Biology, Lomonosov Moscow State University, Moscow, Russia; 2 Mitoengineering Research Institute, Lomonosov Moscow State University, Moscow, Russia; 3 Research Center of Neurology, Russian Academy of Medical Sciences, Moscow, Russia; 4 Faculty of Bioengineering and Bioinformatics, Lomonosov Moscow State University, Moscow, Russia; 5 International Laser Center, Lomonosov Moscow State University, Moscow, Russia; Université Joseph Fourier, France

## Abstract

**Background:**

Many ischemia-induced neurological pathologies including stroke are associated with high oxidative stress. Mitochondria-targeted antioxidants could rescue the ischemic organ by providing specific delivery of antioxidant molecules to the mitochondrion, which potentially suffers from oxidative stress more than non-mitochondrial cellular compartments. Besides direct antioxidative activity, these compounds are believed to activate numerous protective pathways. Endogenous anti-ischemic defense may involve the very powerful neuroprotective agent erythropoietin, which is mainly produced by the kidney in a redox-dependent manner, indicating an important role of the kidney in regulation of brain ischemic damage. The goal of this study is to track the relations between the kidney and the brain in terms of the amplification of defense mechanisms during SkQR1 treatment and remote renal preconditioning and provide evidence that the kidney can generate signals inducing a tolerance to oxidative stress-associated brain pathologies.

**Methodology/Principal Findings:**

We used the cationic plastoquinone derivative, SkQR1, as a mitochondria-targeted antioxidant to alleviate the deleterious consequences of stroke. A single injection of SkQR1 before cerebral ischemia in a dose-dependent manner reduces infarction and improves functional recovery. Concomitantly, an increase in the levels of erythropoietin in urine and phosphorylated glycogen synthase kinase-3β (GSK-3β) in the brain was detected 24 h after SkQR1 injection. However, protective effects of SkQR1 were not observed in rats with bilateral nephrectomy and in those treated with the nephrotoxic antibiotic gentamicin, indicating the protective role of humoral factor(s) which are released from functional kidneys. Renal preconditioning also induced brain protection in rats accompanied by an increased erythropoietin level in urine and kidney tissue and P-GSK-3β in brain. Co-cultivation of SkQR1-treated kidney cells with cortical neurons resulted in enchanced phosphorylation of GSK-3β in neuronal cells.

**Conclusion:**

The results indicate that renal preconditioning and SkQR1-induced brain protection may be mediated through the release of EPO from the kidney.

## Introduction

Many ischemia-induced neurological pathologies including stroke are associated with high oxidative stress. The shift of mitochondrial redox balance may cause a wide spectrum of neurological disturbances from oxidative modification of vital components to selected cell death resulting in neurological deficit or even death of the organism. An anti-oxidative strategy is needed with specific attention to mitochondria both as a source of, and a target for reactive oxygen species (ROS) accompanying oxidative stress associated with ischemia.

The most common and widespread approach to treating brain ischemia uses pharmacologic intervention to prevent or alleviate the deadly effects of stroke. Another approach includes the induction of natural mechanisms of cell protection such as modulation of the immune system or ischemic preconditioning. Recently, pharmacologic treatment of stroke and associated pathologies has demonstrated both appreciable progress and potential that has yet to be realized. Since certain brain pathologies can be attributed to the consequences of oxidative stress, antioxidant treatment may be an effective approach. Mitochondria-targeted antioxidants provide specific delivery of antioxidant molecules to the interior of the mitochondrion, which potentially suffers from oxidative stress more than other cellular compartments [1]–[7]. It was found that mitochondria-penetrating molecules afford a number of beneficial effects, in general protecting the biological system from undesirable consequences of oxidative stress. The latest data suggest that a novel generation of mitochondria-targeted antioxidants (SkQ family) can prevent or correct pathological changes in different tissues (e.g. heart, kidney, brain, and eye) that are characterized by an increased level of ROS [1]–[6], [8]. We recently presented the first evidence of nephroprotective action of substances from the SkQ family, i.e. SkQR1 [10-(6′-plastoquinonyl)decylrhodamine] and SkQ1 [10-(6′-plastoquinonyl)decyltriphenylphosphonium], using a model of kidney ischemia [5], [9], [10]. We also showed the neuroprotective effect of SkQR1 in a few models of brain ischemia [5], [9]. It was demonstrated that SkQR1 is able to induce some components of ischemic tolerance pathways, such as an increase in erythropoietin (EPO) levels and phosphorylation of glycogen synthase kinase 3β (GSK-3 β) in the kidney [10]. Notable, there is evidence that exogenously applied EPO exerts potent neuroprotective effects through preconditioning of the brain [11].

Under normal conditions, EPO controls red blood cell production, and it is mainly produced by the renal interstitial fibroblasts [12]. The regulation of EPO production was shown to involve activation of the hypoxia-inducible transcription factor (HIF), which depends on O_2_/ROS-linkage of its α subunit [13], [14]. In hypoxic cells and tissue, more ROS are generated in mithochondria and HIF-1α protein is stabilized, enabling it to dimerize with HIF-1β and form an active transcription factor. The ROS effect involves in this case inhibition of hydroxylation of a proline residue (HIF-1α P564) and a decrease in the subsequent ubiquitination and degradation of HIF-1α [15], [16].

O_2_/ROS-dependence of EPO expression allows speculation on the existence of a mechanism for indirect neuroprotective action of antioxidants mediated by the main EPO-producing organ which is the kidney. To test this hypothesis we used ischemic preconditioning of the kidney which seems to cause enhanced EPO release [17]. In general, organ preconditioning is a specific strategy which has been developed to protect the organs from ischemia/reperfusion injury. Ischemic preconditioning refers to a phenomenon in which a tissue acquires ischemic tolerance through a set of brief episodes of vascular occlusion [18]. Ischemic preconditioning may be applied directly to the targeted organ or be remote. Remote ischemic preconditioning is a quite novel approach where ischemia followed by reperfusion of one organ protects remote organs due to release of protective signals in the blood, which are received and transformed by a targeted organ [19]. Remote preconditioning was demonstrated for the first time in myocardium by McClanahan et al. [20]. They found that short episodes of ischemia/reperfusion of the kidney protected myocardium from long-term ischemia and reduced infarct size in the heart. In addition, preconditioning of a kidney in order to salvage ischemic myocardium, brief episodes of ischemia/reperfusion of the limb, gut or mesenteric were also used. All of them resulted in a reduced infarct size in myocardium or brain (reviewed in [19]). We note that remote preconditioning of the kidney to salvage the ischemic brain has not been reported yet.

The goal of this work was to track the relations between the kidney and the brain in terms of the amplification of protective mechanisms during SkQR1 treatment and remote renal preconditioning (RRPC). We were seeking for evidence that the kidney can generate signals which induce a tolerance to oxidative stress-associated brain pathologies and activation of defense mechanisms with a primary role for glycogen synthase kinase 3β known as a hub for many protective signaling pathways [21], [22].

## Materials and Methods

### Ethics Statement

Experiments were performed on outbred white male rats (290–350 g). The animals had unlimited access to food and water and were kept in cages with a temperature controlled environment (20±1°C) with light on from 9 AM to 9 PM. The care of experimental animals was performed in strict accordance the guidelines of the “Guide for the Care and Use of Laboratory Animals” (the National Research Council, 1996) and “Euroguide on the accomodation and care of animals used for experimental and other scientific purposes” (FELASA, 2007). All experimental protocols were approved by the Animal Ethics Committees of the Moscow State University (the Protocol Registration number 36). Euthanasia was performed using carbon dioxide in accordance with the 2000 Report of the AVMA Panel on Euthanasia and all efforts were made to minimize suffering of animals. For all surgical procedures rats are anesthetized with i/p injection of 300 mg/kg (12%) chloral hydrate. Additionally, to ensure proper pain relief in the perioperative and postoperative periods we used repeated topical application of a long-acting local anaesthetics bupivacaine ointment. Moribund rats or rats obviously in pain or showing signs of severe and enduring distress was humanely killed. Criteria for making the decision to kill moribund or severely suffering animals, and guidance on the recognition of predictable or impending death were performed in accordance with the Guidelines for Endpoints in Animal Study Proposals (DHHS NIH Office of Animal Care and Use).

### Middle Cerebral Artery Occlusion Model of Focal Ischemia

Middle cerebral artery occlusion (MCAO) surgery or sham operation was performed as previously described [23]. Briefly, rats were anesthetized with i/p injection of 300 mg/kg chloral hydrate. The right common carotid artery was exposed through a midline cervical incision. A heparinized intraluminal silicon-coated monofilament with Ø 0.25 mm was introduced via the external carotid artery into the internal carotid artery to occlude the blood supply to the middle cerebral artery territory. A feedback-controlled heating pad maintained core temperature (37.0±0.5°C) during ischemia supplemented with an infrared lamp until awake. After 60 min of occlusion, the filament was gently pulled out and the external carotid artery was permanently closed by cauterization. In sham-operated rats, the right common carotid artery was exposed and the external carotid artery was electrocoagulated without introducing the filament into the internal carotid artery. SkQR1 (0.5, 1 or 2 µmol/kg) was i/p injected 24 h before MCAO. SkQR1 was synthesized in the Mitoengineering Research Institute and described in details in [10]. Rats were randomly divided into the following groups: (1) sham+VEHICLE (n = 6), (2) MCAO+VEHICLE (n = 12), (3) MCAO+SkQR1 0.5 µmol/kg (n = 13), (4) MCAO+SkQR1 1 µmol/kg (n = 11), (5) MCAO+SkQR1 2 µmol/kg (n = 7). Infarct volume was quantified by analyzing brain MR-images or sections stained with 2% 2,3,5-triphenyltetrazolium chloride (TTC) obtained 24 h after the MCAO as described previously [24]. Brain swelling was also measured in the TTC-stained brain sections or MR-images and calculated using a formula: swelling (edema) = (the volume of the right hemisphere – the volume of the left hemisphere)/the volume of the left hemisphere [25]. Ischemic damage volume for each group was normalized to the mean for the group MCAO+VEHICLE.

### Renal Preconditioning

Rats were anaesthetized with chloral hydrate (300 mg/kg, i.p.). A 2-cm long incision was made on the abdomen. Unilateral renal arteries were clamped by a microvascular clip. Three episodes of ischemia and reperfusion, each comprising 5 min occlusion and 5 min reperfusion, were used to produce preconditioning of a kidney. 24 h after the last episode of preconditioning, the rats were subjected to MCAO as described above. Immediately after renal preconditioning rats were placed in metabolic cages for the urine collection during next 24 h. Rats were allocated into two groups: (6) MCAO (n = 12), (7) MCAO+RRPC (n = 7).

### Bilateral Nephrectomy (BNe)

Rats were anaesthetized with 1% isoflurane in air. After anesthesia, animals were placed on a heating pad to maintain a temperature of 37.0±0.5°C and underwent midline laparotomy with isolation of bilateral renal pedicles. Both kidneys were removed after ligation of the blood vessels and ureter tracts. After 3 h the animals were injected i/p with 1 µmol/kg SkQR1 and 24 h later the rats were subjected to MCAO using isoflurane anesthesia. Rats exposed to MCAO and treated with vehicle were used as controls. Animals were allocated into the following groups: (8) MCAO+VEHICLE (n = 6), (9) MCAO+BNe (n = 9), (10) MCAO+BNe+SkQR1 (n = 8).

### Gentamicin Nephrotoxicity Protocol

Renal failure was induced by administering gentamicin (GM) i/p in a single daily dose of 150 mg/kg over a period of 6 consecutive days. On day 7 the animals were injected i/p with 1 µmol/kg SkQR1 and 24 h later the rats were subjected to MCAO. EPO levels in the daily urine were measured on the 7th day after gentamicin treatments. Rats were randomly divided into three groups: (10) MCAO+GM (n = 10), (11) MCAO+GM+SkQR1 (n = 8), (12) MCAO+GM +RRPC (n = 7).

### Limb-placing Test

The modified version of the limb-placing test consisting of seven tasks was used to assess forelimb and hindlimb responses to tactile and proprioceptive stimulation [26]. The rats were habituated for handling and tested before operation and after the reperfusion for 24 h. For each task, the following scores were used: 2 points, normal response; 1 point, delayed and/or incomplete response; 0 points, no response. Over seven tasks the integral score was evaluated.

### Renal Tubular Epithelium Cell Cultures

Kidneys were excised aseptically from 4-day-old rats, then homogenized and placed in balanced Hank’s solution at pH 7.4. After several washes, the dissociated tissue was placed in 0.1% collagenase and incubated for 20–30 min at 37°C. Cells were sedimented by gentle centrifugation (100 g) for 3 min. The pellet was resuspended in DMEM/F-12 1∶1 containing 10% fetal calf serum (FCS) and seeded in 6-well plate. Cells were cultivated in a CO_2_ (5%) incubator for 1–2 days before the experiments.

**Primary neuronal cultures of cerebral cortex** were obtained from embryos (16–18 days) of outbred white rats. Cultures were prepared according to Brewer [27] with the following modifications: cerebral cortex was dissected, meninges were removed and tissue was incubated for 15 min in trypsin/EDTA (0.05/0.02% wt/vol in PBS) at 37°C; the cultures were rinsed twice with PBS and once with dissociation medium (modified Eagle’s medium with 10% fetal calf serum, 10 mM HEPES, 100 U penicillin streptomycin/ml, 2 mM L-glutamine); dissociated by Pasteur pipette in dissociation medium, pelleted by centrifugation (210 *g* for 2 min at 21°C) and redissociated in Neurobasal medium with supplemental B27-AO (Invitrogen, Paisley, UK), 100 U penicillin streptomycin/ml, 0.5 mM L-glutamine, 25 µM glutamate. Cell suspension was applied to poly-L-lysine-coated 75 cm^2^ flasks or to 24-well plate with round cover slips. Cultures were kept at 36.5°C and 5% CO_2_ and after 4 days *in vitro* twice a week the one-half of the medium was replaced by a basal medium without glutamine. The cultures were used for experiments after 8 days.

**Astroglial cell cultures** were prepared according to a modified method described by McCarthy and de Vellis [28]. In brief, meninges from cortices of newborn outbred white rats were removed and the tissue was mechanically dissected and digested in trypsin/EDTA solution (0.05% trypsin, 0.02% EDTA) at 37°C for 15 min. After digestion, the tissue was washed twice in PBS followed by a mechanical dissociation with a pipette in MEM. The dissociated cells were seeded in 75 cm^2^ flasks (2 brains/flask). Cells were grown in MEM (10% FCS, 1% penicillin/streptomycin, 2 mM L-glutamine, 10 mM HEPES). After 8–10 days the cultures were reseeded in subcultures or used for experiments. SkQR1 50 and 100 nM was supplemented directly to the cultivated medium for 24 h. After incubation with SkQR1 cells were immediately used for Western blotting.

### Co-culture of Renal Tubular Epithelial Cells with Primary Neuronal Culture of Cerebral Cortex

After 2–3 days of cultivation, SkQR1 (200 nM, final) was added to the kidney cells culture followed by washing with PBS and subsequent addition of Neurobasal medium with supplemental B27-AO, 100 U penicillin streptomycin/ml, 0.5 mM L-glutamine. Cover slips with attached primary neuronal cultures of cerebral cortex were transferred to kidney cell culture for 24 h. Other cover slips were transferred to untreated kidney cell culture.

### Immunocytochemistry of Primary Neuronal Cultures

Cells were washed in PBS, fixed for 15 min in 4% formaldehyde with PBS at 4°C, and permeabilized in PBS containing 0.02% Triton X-100 for 15 min followed by blocking in PBS with 1% bovine serum albumin (PBS-BSA) for 60 min. P-GSK-3β was detected using mouse monoclonal antibodies (Cell Signaling, USA) diluted 1∶200 in PBS-BSA at 4°C overnight. After three 15-min rinses in PBS-BSA, cells were incubated for 1 h in FITC-conjugated anti-mouse IgG (Sigma Aldrich, USA) with 1∶100 dilution. The cover slips with cells attached were washed, placed on microscope slides with a mounting medium and sealed beneath cover slips with nail varnish. Cells were imaged with an LSM510 inverted confocal microscope (Carl Zeiss Inc., Jena, Germany). Images were processed using ImageJ software (NIH, Bethesda, MD, USA).

### Urine Samples, Erythropoietin Immunoassay

The rats were injected i/p with 1 and 2 µmol/kg SkQR1 or RRPC was used immediately after whose rats were placed in metabolic cages for the urine collection during next 24 h. Briefly, 5 ml of urine were filtered using Steriflip microfiltration units after addition of 0.5 ml of 3.75M Tris (pH 7.4). For concentrating, the centrifugation with Centricon YM 30 ultrafiltration units was performed. The resulting retentates of about 50 µL were stored at –20°C until analysis. The final retentate was assayed for EPO level by ELISA Quantikine® assay according to the manufacturer’s instructions (R&D Systems, USA).

### Hematology Analysis

A blood samples were taken from a tail vein on the 12 and 24 h after i/p treatment with 2 µmol/kg SkQR1 for further analysis. Red blood cels, hemoglobin and hematocrit were determined using the PCE-90Vet veterinary hematology analyzer.

### Western Blot Analysis

Samples of brain tissue homogenates were loaded onto 15% Tris–glycine polyacrylamide gels (50 µg of total protein per lane). After electrophoresis, gels were blotted onto PVDF membranes (Amersham Pharmacia Biotech, UK). Membranes were blocked with 5% (wt/vol) non-fat milk in PBS with 0.1% (vol/vol) Tween 20 and subsequently incubated with primary antibodies: rabbit polyclonal anti-P-GSK-3β with 1∶1000 dilution (Cell Signaling, USA), mouse monoclonal anti-total-GSK-3β with 1∶1000 dilution (Cell Signaling, USA) or rabbit polyclonal anti-EPO with 1∶1000 dilution (Santa Cruz Biotechnology, USA). Membranes were then treated with secondary antibodies with 1∶10000 dilution (anti-rabbit or anti-mouse IgG conjugated with horseradish peroxidase, respectively). Specific bands were visualized using ECL Plus Western blotting kit (Amersham Pharmacia Biotech, UK). After scanning, the density of the resulting staining was measured for each band using ImageJ software (NIH, Bethesda, MD, USA).

### Analysis of SkQR1 Accumulation in Kidney and Brain

Rats were injected i/p with 1 µmol/kg SkQR1 and kidneys and brains were excised after 3 and 24 h, fixed in 4% formaldehyde with PBS and sliced using a VibroSlice microtome (World Precision Instruments) into 100 µm thick sections. Slices were imaged with a LSM510 inverted confocal microscope (Carl Zeiss Inc., Jena, Germany) with excitation at 543 nm and emission collected at 560–590 nm. As a negative control organs from untreated animals were used.

### Statistics

Statistical analyses were performed using STATISTICA 7.0 for Windows (StatSoft, Inc.). All data were presented as means ± standard error of means (SEM) apart from the neurological deficit scores are expressed as median ± interquartile ranges, the 25th to 75th percentile are shown in the parentheses. Variance homogeneity was assessed with Levene’s test. Statistical differences between groups in the data of infarct volume, brain swelling, immunocytochemistry, densitometry, hematological parameters and ELISA were analyzed using one-way ANOVA with Tukey’s post hoc test or *t* tests for independent samples. Statistical differences in limb-placing tests between groups were analyzed using Kruskal-Wallis test with the Mann–Whitney *u*-test with Bonferroni correction post hoc, and analyzed with Mann–Whitney test when comparing between two groups. Values for P<0.05 were considered statistically significant.

## Results

### Mortality and Adverse Effects

Out of 148 rats undergoing MCAO surgery procedure, 26 died within 24 h after operation, with the main cause being hemorrhage and severe edema. Six MCAO rats were excluded from the study because the animals did not show sufficient behavioral deficit in the limb-placing test (with the score exceeding 10 points) and MRI could not reveal the infarct areas. A typical infarct was considered to comprise extensive cortical and striatal damage and also variable partial damage to the hypothalamus and amygdale outside the vascular territory of the middle cerebral artery.

### SkQR1 and RRPC Protects against Ischemic Brain Injury

**Figure 1:**
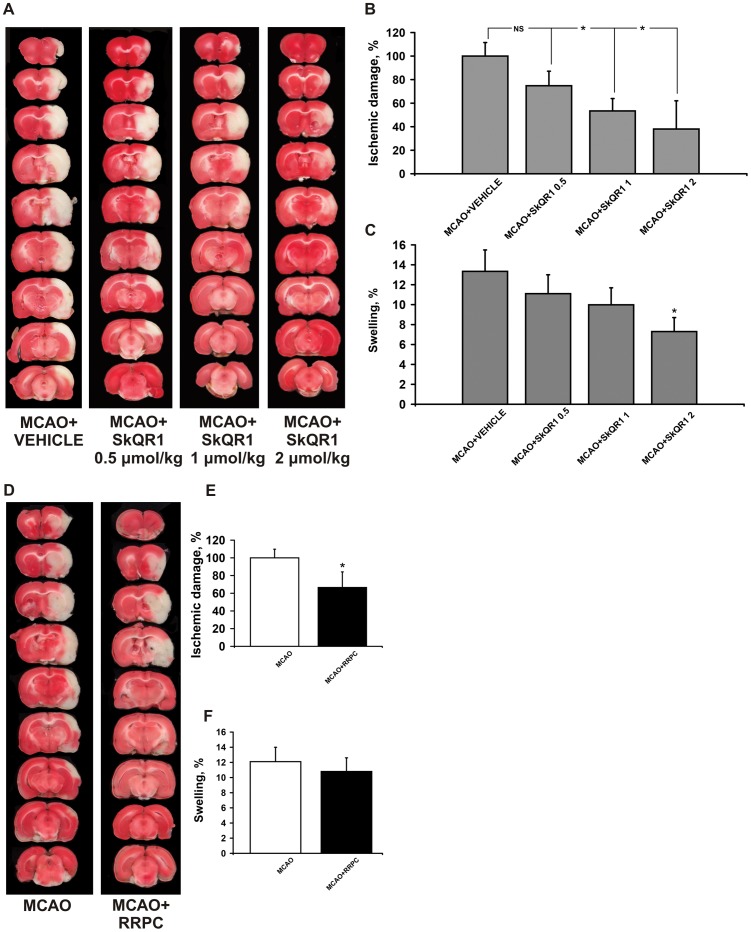
The treatment with SkQR1 and RRPC starting before the MCAO protects the ischemic injured brain. Rats in SkQR1-pretreated groups (A, B, C) were given i/p SkQR1 (0.5, 1, 2 µmol/kg) or (D, E, F) subjected to remote renal preconditioning (RRPC) 24 h prior to ischemia. (A, D) Representative photomicrographs from coronal brain sections (2 mm thick, from rostral (top) towards caudal (bottom)) stained with TTC 24 h after reperfusion. Lack of red staining indicates infarction. (B, E) Infarct volume and (C, F) brain edema (swelling) were measured in the TTC-stained brain sections. * Denotes significantly different from the MCAO+VEHICLE or MCAO groups (p<0.05) (One-way ANOVA, followed by Tukey’s post hoc analysis or *t* tests for independent samples).

First, we examined the protective effects of various doses of SkQR1 on ischemic brain injury after administration of SkQR1 24 h before MCAO. When compared with the vehicle group, infarct volume was reduced significantly after treatment with 1 and 2 µmol/kg SkQR1 (p<0.05) (Fig. 1A, B). The treatment with 2 µmol/kg SkQR1 also significantly reduced brain swelling (p<0.05) (Fig. 1C). There were no statistically significant differences on infarct volume (p = 0.076) and swelling after treatment with 0.5 µmol/kg SkQR1. The treatment with SkQR1 at a dose 0.5, 1 and 2 µmol/kg also improved functional recovery as measured by neurologic deficit scores (Table 1). Accordingly, RRPC-treated rats 24 h before MCAO also significantly reduced infarct size, but not the brain swelling. RRPC was able to induce a robust protection of the brain in experimental stroke with infarct volume reduced to 34% of control and improvement of the total score in the limb-placing task (Fig. 1D–F; Table 1).

**Table 1:** Neurological state^a^.

Experements	Group	Score
**The SkQR1 study**	Baseline	14 (14–14)
	Sham+ VEHICLE	13.5 (13.25–14)
	MCAO+ VEHICLE	1 (1–2)
	MCAO+ SkQR1 0.5 mol/kg	5 (3–7)*
	MCAO+ SkQR1 1 µmol/kg	5.5 (4.5–7.5)*
	MCAO+ SkQR1 2 µmol/kg	6.5 (3.75–9.25)*
**Renal preconditioning**	MCAO	3 (2–3)
	MCAO+ RRPC	11 (10.5–11)*
**Bilateral nephrectomy**	MCAO	3 (3–4.5)
	MCAO+ BNe	5 (3–6)
	MCAO+BNe+SkQR1 1 µmol/kg	3,5 (2.75–4.25)
**Gentamicin-induced**	MCAO+GM	4 (3–9)
**renal failure**	MCAO+ GM+SkQR1 1 µmol/kg	3 (2.75–4)
	MCAO+ GM+RRPC	4 (2.5–5.5)

a The neurological state scores are recorded in a blind fashion and expressed as median and interquartile ranges, the 25th to 75th percentile are shown in the parentheses. Baseline represents neurological state of intacte rats. *Denotes significant from the score at 24 h after the MCAO compared to MCAO+ VEHICLE or MCAO groups (p<0.05) (Kruskal-Wallis test with the Mann–Whitney *u*-test with Bonferroni correction post hoc or Mann–Whitney test when comparing between two groups).

### SkQR1 and RRPC Provides Some Features of Ischemic Tolerance

**Figure 2:**
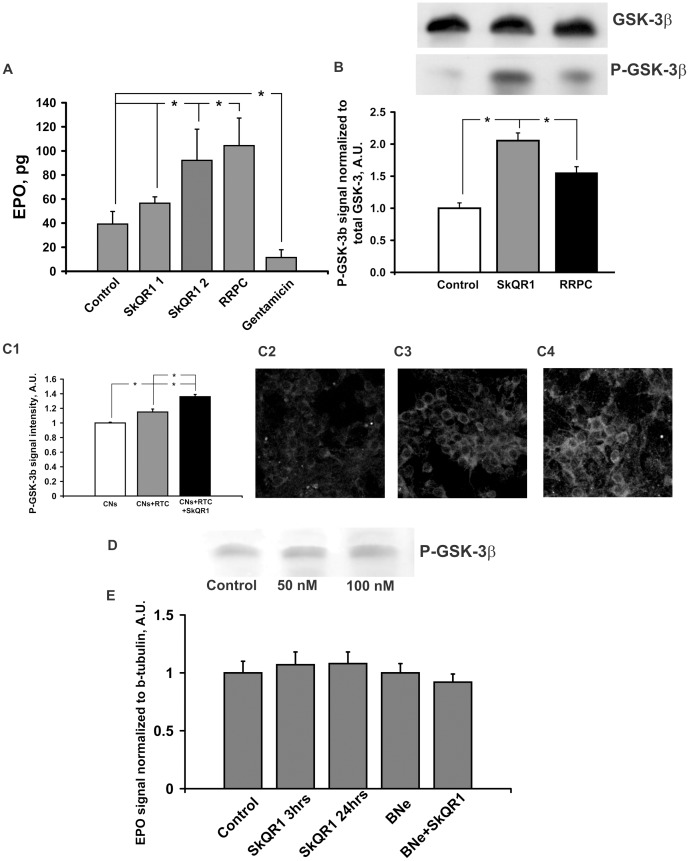
SkQR1 and RRPC provide some features of ischemic tolerance. (**A**) Changes in erythropoietin (EPO) level in the daily urine. 1 or 2 µmol/kg SkQR1 was injected i/p 24 h before urine collection. Rats were subjected to RRPC for 24 h and gentamicin for 6 days prior to urine collection. (**B**) Detection of phosphorylated glycogen synthase kinase-3β (P-GSK-3β) in the total brain tissue. Representative Western blots with corresponding densitometry averaged over 6 blots are shown. Band densities were normalized to the density of total GSK-3β band. 1 µmol/kg SkQR1 was injected i/p 24 h before excising the brain. (**C1–4**) Detection of P-GSK-3β in cultured cortical neurons (CNs) after co-culturing with renal tubular cells (RTC) treated with 250 nM SkQR1 for 1 h before 24 h of co-culturing (C1); control CNs (C2); co-culture of CNs and RTC (C3); co-culture of CNs with RTC primed with SkQR1 (C4). (**D**) Detection of P-GSK-3β in the cultural glial cells treated with 50 or 100 nM SkQR1 for 24 h. (**E**) Detection of EPO in the entire brain tissue. Densitometry of Western EPO spots averaged over 6 blots is shown. 1 µmol/kg SkQR1 was injected i/p 3 or 24 h before excising the brain. * Denotes significantly different from the control group (p<0.05) (One-way ANOVA, followed by Tukey’s post hoc analysis or *t* tests for independent samples).

In the experiments presented above we observed an essential neuroprotective potential of a mitochondria-targeted compound, SkQR1, and RRPC. To gain insight into the mechanisms of neuroprotection afforded by SkQR1 and RRPC, we measured the concentrations of two components of anti-ischemic signaling systems, specifically EPO and P-GSK-3β. Single i/p injection of 1 and 2 µmol/kg SkQR1 caused 45 and 235% rise of EPO level in the daily urine, respectively, and there was 265% rise of EPO in urea after RRPC (Fig. 2A). After 3 or 24 h of i/p injection of 1 µmole/kg SkQR1 the EPO level in the brain tissue was not changed as well as in the brain of nephrectomized animals after 24 h of injection (Fig. 2E). Furthermore treatment with SkQR1 at dose 2 µmol/kg increased number of red blood cells and hemoglobin level 24 h after administration (Table 2). The level of phosphorylated GSK-3β in the brain homogenates (P-GSK, enzymatically inactive form) reached 210% and 160% vs the baseline 24 h after i/p SkQR1 injection (1 µmol/kg) or RRPC respectively (Fig. 2B). Remarkably, we detected the increase in the level of P-GSK-3β in primary neuronal cultures of cerebral cortex after their co-culturing with renal tubular epithelial cells and a larger increase when co-cultivated renal cells were primed with 250 nM SkQR1 for 1 h before 24 h of co-culturing in the SkQR1-free cultivation medium (Fig. 2C). Meanwhile, the incubation of primary astroglial cell cultures with 50 or 100 nM SkQR1 did not cause an increase of P-GSK-3β level (Fig. 2D).

**Table 2:** Hematological parameters^a^.

Group	RBC, 10^12^/L	HGB, g/L	HCT%
Vehicle 12 h	8.8±0.3	132±6.4	50.9±2.1
SkQR1 12 h	9,3±0.3	136,9±1.9	52.8±2
Vehicle 24 h	8.1±0.4	126.5±6,8	47.7±2.7
SkQR1 24 h	9.5±0.2*	146.4±5.4*	52.3±2.2

a Hematological parameters changes after treatment with 2 µmol/kg SkQR1 for 12 h: Vehicle (n = 8) and SkQR1 (n = 8); for 24 h: Vehicle (n = 6) and SkQR1, (n = 7). RBC, red blood cells; HGB, hemoglobin; HCT, hematocrit.

* Denotes significantly different from the vehicle group (p<0.05) (*t* tests).

### The Role of the Kidney in Neuroprotection

**Figure 3:**
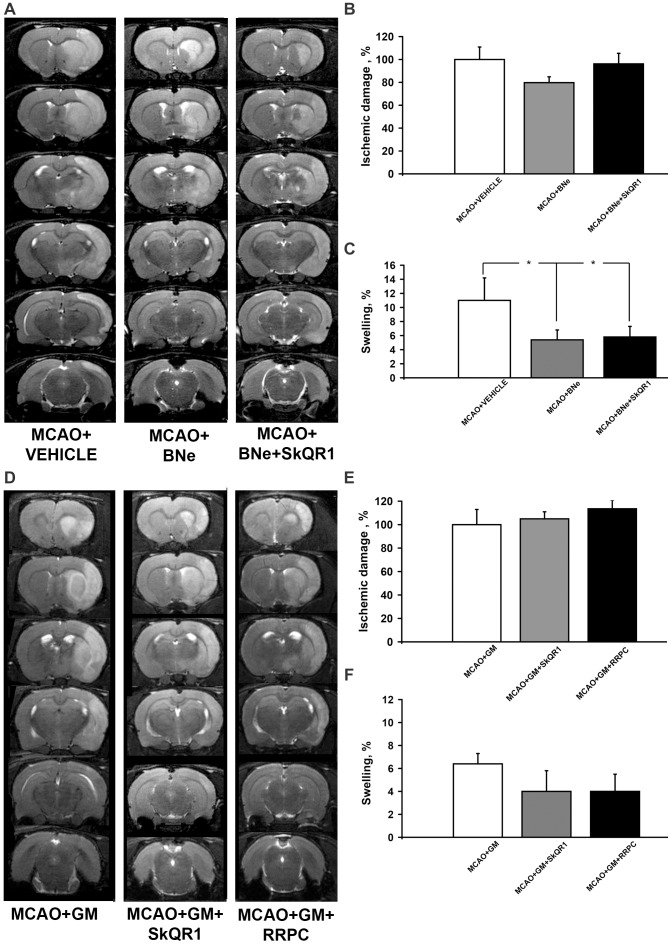
Bilateral nephrectomy and gentamicin pretreatment abolish neuroprotective action of SkQR1 and RRPC. The effects of SkQR1 after bilateral nephrectomy on the brain infarct volume and brain swelling of rats exposed to MCAO are shown in (A, B) and (C) correspondingly. Nephrotoxic gentamicin pretreatment shows similar abrogation of beneficial effects of SkQR1 and RRPC on the brain infarct volume (D, E) and brain swelling (F) of rats exposed to MCAO. (A, D) Representative T2-weighted MR-images from coronal brain sections (0.5 mm thick, from rostral (top) towards caudal (bottom)) obtained 24 h after reperfusion. Hyperintensities regions refer to ischemic areas. The evaluation of the brain damage area and brain swelling were done by using MRI with analysis of T2-weighted images.

We studied a potential role of the kidney-mediated neuroprotection using following tools: 1) the ischemic preconditioning of the kidney; 2) pharmacological preconditioning induced by SkQR1, 3) bilateral nephrectomy and, 4) gentamicin-induced renal failure. As judged by the brain infarct volumes and the neurologic deficit of animals, advanced bilateral nephrectomy abolished a protective effect of SkQR1 (1 µmol/kg) injected 24 h before MCAO (Fig. 3A, B; Table 1). Bilateral nephrectomy caused significantly decreased swelling of brain in all groups that can be explained by the changes of the blood osmolytes after removal of the kidney (Fig. 3C). In addition, we ran experiments with antibiotic gentamicin known for its nephrotoxicity as a side effect when applied. A chronic gentamicin supplement (150 mg/kg daily for 6 days) caused a severe renal failure with creatinine being 218 µM and urea being 34 mM. In gentamicin-treated animals, we observed a significant drop of EPO level in daily urine (Fig. 2A). RRPC and SkQR1 (1 µmol/kg) injected 24 h before MCAO failed to attenuate either increased infarct size/brain swelling or behavior impairment in ischemic animals with gentamicin-induced renal failure (Fig. 3D–F; Table 2). Control experiments showed that gentamycin treatment and bilateral nephrectomy alone did not cause neurological disturbances basing on same tests (not shown).

### Kinetics of SkQR1 Accumulation in Kidney and Brain

Further, we analyzed accumulation of SkQR1 in kidney and brain by the distribution of fluorescence using tissue sections sampled 3 and 24 h after i/p injection of SkQR1 to the rat. Earlier, we demonstrated that 10 min after i/p injection of SkQR1 the fluorescence in the tissue of kidney was not observed and only appeared starting at 60 min after injection, confined to glomerular regions with further redistribution over tubules [5].

**Figure 4:**
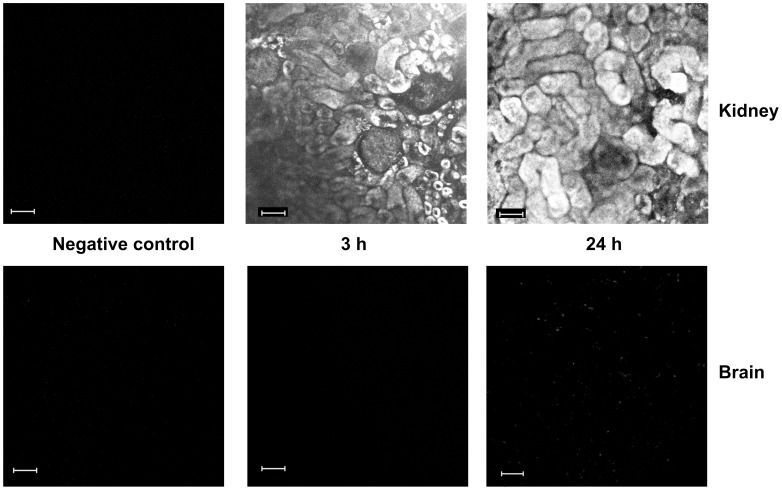
SkQR1 accumulates in kidney but not in brain. SkQR1 retention and distribution over kidney and brain compartments 3 and 24 hrs after i/p injection of 1 µmol/kg SkQR1. Confocal microscopy of tissues slices. As a negative control, organs from untreated animals were also analyzed. Bar, 50 µm.

In this study, we analyzed SkQR1 tissue distribution 3 and 24 h after injection in the dose of 1 µmol/kg. We found that while in the kidney fluorescence of SkQR1 was visible 3 h after injection and remained detectable up to 24 h after injection, brain tissue did not show fluorescence attributable to SkQR1 (Fig. 4) except scattered spotted fluorescence apparently due to a minimal accumulation in the blood vessel cells at 24 h. Possibly, this difference is due to low permeability of the blood brain barrier for SkQR1.

## Discussion

Pharmacological correction of pathologies related to acute failure of the cerebral blood circulation remains one of the main problems of experimental and clinical neurology and neurosurgery, which is a reason for creation of adequate model for the study of ischemia and the development of novel drugs affording neuroprotection. The MCAO model is most relevant to clinical forms of ischemic brain injury [23], [29] and is widely used to test potential neuroprotectors. Antioxidants could be considered as potential neuroprotectors in pathologies mediated by oxidative stress. However, until now there were no convincing clinical data on the ability of antioxidants to prevent ischemic damage [30] which is partially explained by the problem of a delivery of these drugs to the sites of ROS generation at the right moment and in sufficient amount to quench oxidative stress [31]. In this context, the most promising would be a new trend to use antioxidants specifically targeted to mitochondria for the therapy of diseases associated with oxidative stress[1]–[7], [32]–[34]. It has been already shown that these compounds, specifically plastoquinone-based ones, such as SkQ1 and SkQR1 improve kidney and heart functioning after ischemia and protect neurons from cell death after compression of the brain or skull-brain damage [5], [35]. Earlier, a positive anti-ischemic effect of another mitochondrial coenzyme Q-based antioxidant, MitoQ, was demonstrated in a heart ischemia model [32]; however, this compound failed to protect the brain from ischemic damage [33].

We extended the possible range of nephroprotective properties of penetrating cations of the SkQ family (in particular, SkQR1), showing that ischemia-driven kidney pathologies, myoglobinuria and pyelonephritis can be prevented (and seemingly reversed) by SkQR1 [10], [36]. Renal protection from these pathologies was demonstrated to correlate with the ability of the kidney to produce EPO. Such induced protective EPO production was not unique since similar induction was demonstrated for vitamin A or N-acetyl-cysteine, as well as the ability of antioxidants to diminish the effect of H_2_O_2_ to lower EPO production has been also shown [37]–[39]. On healthy volunteers it has been shown that oral admistration N-acetyl-cysteine increases EPO concentration in the plasma [40]–[42].

Currently, it is widely accepted that EPO is a universal intercellular messenger of ischemic tolerance of the brain [11]. In addition, intercellular communication as a route of protective signaling has, in large part, been revised and updated. This gap has been partially filled by Ruscher and co-autors [11] by demonstrating that HIF-1 is activated rapidly by hypoxia in astrocytes and after this activation the astrocytes express and release EPO. EPO was found to activate the neuronal EPO receptor and, subsequently, JAK-2 and thereby PI3K. In this pathway, downstream of these kinases there are two other kinases, Akt and GSK-3b with GSK-3b being a substrate of Akt [43]. The main regulatory mechanism of these enzymes is mediated by phosphorylation but while Akt is activated GSK-3b is inhibited by phosphorylation. Further, PI3K deactivates BAD via Akt-mediated phosphorylation and thus may inhibit hypoxia-induced neuronal apoptosis. Activation of a wide spectrum of brain protective signaling pathways is associated with phosphorylation and inhibition of a discrete pool of GSK-3β. GSK-3β located in the vicinity of the mitochondrial permeability transition pore complex is believed to be a key enzyme that the protective signaling pathways converge on [44]. The key role of GSK-3β in preconditioning pathways affording organ protection was first discovered for the heart [21] and confirmed for other organs, e.g., for kidney [22]. GSK-3β activation was shown to promote neuronal apoptosis, particularly through the inducible release of cytochome *c*, while kinase inhibition may enhance neuroprotection [45]. In general, this kinase receives signals from upstream signaling cascade elements and enhances tolerance of cells to oxidative injury (reviewed in [46], [47]). However, after i/p injection of SkQR1 we could not detect induction of EPO production in the brain, although this compound significantly diminished the size of the brain ischemic damage, lowered neurological deficit after focal brain ischemia, and inhibited proapoptotic enzyme GSK-3β, increased EPO in the urine and both the number of erythrocytes and hemoglobin in the blood as well. Thus, i/p introduction of SkQR1 leads to a rise of renal EPO production but not in the brain, however, affording brain protection related to inhibition of GSK-3β. These observations could be explained by low permeation of SkQR1 through the blood-brain barrier, since we were not able to detect SkQR1 in brain tissues by confocal microscopy. At the same time, penetration and accumulation of SkQR1 in kidney tissues were appreciable. Co-cultivation of neurons and renal cells primed with SkQR1 also results in inhibition of neuronal GSK-3β. At the present, in a number of studies it has been demonstrated that systemic administration of exogenous EPO is neuroprotective [48]. In addition, remote renal preconditioning-induced cardioprotection [49] was explained by an activation of a signaling preconditioning mechanism mediated by EPO. It was shown that cardioprotective effects of RRPC were not observed in rats with renal failure induced by gentamicin [17].

However, similar studies on the role of the kidney in brain protection have not been performed yet. Our experiments obviously demonstrate the role of EPO in providing ischemic tolerance, thus emphasizing existing mechanisms of inter-organ signaling. We found that transient renal ischemia causes elevation of the EPO level in this tissue and in the urine and alleviate subsequent ischemic damage of the brain. It is worth highlighting that for animals either with nephrectomy or exposed to nephrotoxic antibiotic gentamicin SkQR1 was not neuroprotective. Unfortunately, our efforts to detect the changes of EPO level in the blood failed either due to extremely low basal levels of EPO in the blood or that EPO release occurs in a pulse mode which is hard to detect. However, EPO appearance in the urine gives a strong support of an EPO rise (may be temporarily) in the blood.

Currently, rather controversial results have been received in a number of clinical trials on therapeutic use of EPO in ischemic heart and brain. For example, some studies have shown that EPO increased incidence of microvascular obstruction [50] and EPO treatment was associated with higher rates of adverse cardiovascular events [51]. In another trial after epoetin β introduction immediately after reperfusion the incidence of microvascular obstruction was decreased and associated with transient favorable effects on left ventricular volume and function [52]. Possible failures might be explained by high doses of EPO used in the trial and/or its unfavorable combination with thrombolitic therapy. The German Multicenter EPO Stroke Trial, which investigated safety and efficacy of EPO treatment in ischemic stroke, formally declared a negative result [53]. However, exploratory subgroup analysis, revealed that patients not receiving thrombolysis most likely benefited from EPO during clinical recovery [54]. In addition, Bergmann and colleagues showed that low-dose epoetin-β treatment following percutaneous coronary intervention is safe and feasible, and has possible beneficial effects on global ejection fraction and measures of exercise capacity [55]. Thus, pharmacologic or remote preconditioning directed to elevation of a basal EPO level might be a more preferable therapeutical strategy.

Ultimately, our data demonstrate that the neuroprotective effect of SkQR1 and remote renal preconditioning can be explained by stimulation of renal production of EPO which is an essential mediator of neuroprotection and that interorgan signaling plays a critical role for initiation of brain ischemic tolerance.
